# Involvement of the ipsilateral-to-the-pain anterior–superior hypothalamic subunit in chronic cluster headache

**DOI:** 10.1186/s10194-023-01711-0

**Published:** 2024-01-11

**Authors:** Stefania Ferraro, Anna Nigri, Maria Grazia Bruzzone, Jean Paul Medina Carrion, Davide Fedeli, Greta Demichelis, Luisa Chiapparini, Giuseppe Ciullo, Ariosky Areces Gonzalez, Alberto Proietti Cecchini, Luca Giani, Benjamin Becker, Massimo Leone

**Affiliations:** 1 School of Life Science and Technology, MOE Key Laboratory for Neuroinformation, University of Electronic Science and Technology of China, Chengdu, China; 2 Center of Psychosomatic Medicine, Sichuan Provincial Center for Mental Health, Sichuan Provincial People’s Hospital, University of Electronic Science and Technology of China, Chengdu, China; 3 Department of Neuroradiology, Fondazione IRCCS Istituto Neurologico Carlo Besta, Via Celoria 11, Milan, Italy; 4 Radiology Unit, Fodazione IRCCS Policlinico San Matteo, Pavia, Italy; 5 Department of Medicine and Surgery, University of Parma, Parma, Italy; 6 Faculty of Technical Sciences, University of Pinar del Río “Hermanos Saiz Montes de Oca”, Pinar del Río, Cuba; 7 Department of Neuroalgology, Fondazione IRCCS Istituto Neurologico Carlo Besta, Milan, Italy; 8 Department of Neurology, Fondazione Maugeri, IRCCS, Milan, Italy; 9 State Key Laboratory of Brain and Cognitive Sciences, The University of Hong Kong, Hong Kong, China; 10 Department of Psychology, The University of Hong Kong, Hong Kong, China

**Keywords:** MRI, Chronic cluster headache, Hypothalamus, Paraventricular nucleus, Preoptic area

## Abstract

**Background:**

Despite hypothalamus has long being considered to be involved in the pathophysiology of cluster headache, the inconsistencies of previous neuroimaging studies and a limited understanding of the hypothalamic areas involved, impede a comprehensive interpretation of its involvement in this condition.

**Methods:**

We used an automated algorithm to extract hypothalamic subunit volumes from 105 cluster headache patients (57 chronic and 48 episodic) and 59 healthy individuals; after correcting the measures for the respective intracranial volumes, we performed the relevant comparisons employing logist regression models.

Only for subunits that emerged as abnormal, we calculated their correlation with the years of illness and the number of headache attacks per day, and the effects of lithium treatment. As a post-hoc approach, using the 7 T resting-state fMRI dataset from the Human Connectome Project, we investigated whether the observed abnormal subunit, comprising the paraventricular nucleus and preoptic area, shows robust functional connectivity with the mesocorticolimbic system, which is known to be modulated by oxytocin neurons in the paraventricular nucleus and that is is abnormal in chronic cluster headache patients.

**Results:**

Patients with chronic (but not episodic) cluster headache, compared to control participants, present an increased volume of the anterior–superior hypothalamic subunit ipsilateral to the pain, which, remarkably, also correlates significantly with the number of daily attacks. The post-hoc approach showed that this hypothalamic area presents robust functional connectivity with the mesocorticolimbic system under physiological conditions. No evidence of the effects of lithium treatment on this abnormal subunit was found.

**Conclusions:**

We identified the ipsilateral-to-the-pain antero-superior subunit, where the paraventricular nucleus and preoptic area are located, as the key hypothalamic region of the pathophysiology of chronic cluster headache. The significant correlation between the volume of this area and the number of daily attacks crucially reinforces this interpretation. The well-known roles of the paraventricular nucleus in coordinating autonomic and neuroendocrine flow in stress adaptation and modulation of trigeminovascular mechanisms offer important insights into the understanding of the pathophysiology of cluster headache.

**Supplementary Information:**

The online version contains supplementary material available at 10.1186/s10194-023-01711-0.

## Background

Cluster headache (CH) is characterized by a distinctive pattern of cyclic recurrence of short-lasting unilateral excruciating craniofacial pain accompanied by trigeminal autonomic symptoms ipsilateral to the head pain such as rhinorrhea, eyelid edema and miosis/ptosis (Arnold, 2018). The exact pathophysiological mechanisms of this neurological disorder and its chronicity, which occurs in 10–20% of patients [40], remain to be elucidated [45]. However, the cyclic nature of the disease (circannual recurrence of cluster periods and circadian onset of attacks) and the associated neuroendocrinological abnormalities have led to hypothesize the involvement of the hypothalamus [32], thus reconceptualizing CH as a central disorder and shifting the focus of the research from peripheral mechanisms (e.g., irritation of trigeminal fibers in the cavernous sinus [19] to central mechanisms.

The first direct evidence of the validity of the central pathophysiological hypothesis and the possible involvement of the hypothalamus was provided by the landmark PET neuroimaging study by May et al. [42, 43], which showed robust activity in the posterior hypothalamic area ipsilateral-to-pain during nitroglycerin-induced attacks in patients with the chronic form of CH. Although the exact localization of this activity has been debated [54], subsequent studies confirmed hypothalamic activity also in spontaneous attacks in both chronic (cCH) and episodic CH (eCH) [47, 57]. Further strengthening the hypothalamic hypothesis, May et al. [41, ], employing voxel-based morphometry (VBM), also demonstrated a volumetric increase of the same brain region observed in their previous functional study. However, possibly because of the methodological limitations of this approach [71], some subsequent VBM investigations did not confirm these findings [2, 48, 69] also when employed in relatively large samples of episodic and cCH patients [39, 48, 69]. However, that structural alterations may characterize the hypothalamus in CH was again highlighted by a study employing manual segmentation [4], which showed increased volumes of bilateral anterior regions of this structure in different CH forms (chronic, episodic, and probable CH). Nevertheless, the observation of bilateral hypothalamic changes is challenged by the typically unilateral clinical features of CH attacks, which instead suggest an ipsilateral pattern, at least in critical structures. Manual segmentation can be indeed affected by inter- and intra-rater variability [7], particularly when, as in the case of the hypothalamus, it is applied to small and low-contrast magnetic resonance imaging (MRI) structures. Further complicating the picture, a recent study [31] employed a newly developed and state-of-the-art algorithm to segment the hypothalamus and its subunits [7] without showing any alterations in episodic CH patients.

The inconsistencies of previous results, probably due to methodological limitations, together with the lack of knowledge of the precise hypothalamic areas involved, limit a solid interpretation of the hypothalamus' role in CH conditions.

Indeed, despite more than 20 years of studies, it is still unclear whether there are macroscopic hypothalamic changes in CH patients, whether they are bilateral or not, whether they are more typical of chronic or episodic CH patients, and whether they are linked to clinical variables (e.g., years of disease, number of attacks per day). These aspects are crucial to clarify: indeed, it would be possible to define whether the supposed hypothalamic abnormalities are a trait or a state of CH patients (if present in all CH patients or if present only in CH patients in-bout and in chronic CH patients), or whether they are related to chronic conditions (if present only in chronic CH patients). No less important is the need of determining with relative precision the hypothalamic nuclei involved in the pathophysiology of CH, trying also to fill the gap with clinical and preclinical studies. In this regard, if the first studies showed functional and anatomical abnormalities localized in the posterior section of the ipsilateral-to-the pain hypothalamus in chronic CH patients [41–43], more recently, the study from Arkink (2016) suggested a bilateral involvement of the suprachiasmatic nucleus and of the paraventricular nucleus (PVN) of the hypothalamus.

The most prominent features of CH attacks can account for the abnormality of even the morphological nature of the suprachiasmatic nucleus, the endogenous biological clock [50]. Nevertheless, the PVN is now known to play a pivotal role in regulating circadian rhythms in metabolism and endocrine functions [28]. In addition, PVN neurons were shown to project to the superior salivary nucleus, which is involved in the autonomic phenomena of CH attacks [52, 68] and to the caudal spinal trigeminal nucleus (Sp5C) [52] as well as being critical in orchestrating stress responses [13]. These observations make the PVN the most plausible hypothalamic key player in the pathophysiology of CH, particularly as a regulator of trigeminal activity.

In our study, we ought to define the precise hypothalamic areas involved in CH pathophysiology, identifying volume abnormalities of hypothalamic nuclei grouped in subunits according to the subdivision proposed by Makris et al. [35] and overcoming the main methodological problems. To this aim, we 1) analyzed a large sample of CH patients (105 participants), including both chronic and episodic forms (in-bout and out-of-bout), 2) employed the state-of-the-art algorithm for automatic segmentation of hypothalamic subunits [7], already used in Lee’s study (2022) and the *residual method* for correcting the measurements obtained for brain size [55], 3) used various statistics to distinguish possible biases induced by physiological lateralization effects from genuine abnormalities.

Remarkably, we characterized the observed abnormalities with respect to the major clinical variables.

Finally, using a post-hoc approach, after identifying the antero-superior subunit as a crucial area of abnormality in cCH patients, we examined the functional connectivity between the subunit and the mesocorticolimbic system under physiological conditions employing the 7 T resting-state functional MRI (rs-fMRI) public dataset from healthy participants of the Human Connectome Project [56, 64]. Indeed, data from the literature indicate the existence of robust interactions between the paraventricular nucleus (located in the abnormal subunit) and the mesocorticolimbic system [5, 12, 24], which is functionally and anatomically altered in cCH patients [14]. A common but crucial challenge inherent in many studies investigating brain morphology and function in patient populations is the effect of medications. Ethical considerations discourage researchers from asking patients to discontinue medications prior to MRI, especially if these medications control painful states such as CH attacks. In line with this ethical approach, we did not ask participants in this study to discontinue the use of prescribed drugs, although we did try to control the effect of lithium, which is known to induce increases in some brain areas [1, 22, 37, 65, 70].

## Materials and methods

### Hypothalamic subunits in CH patients

The considered MRI data were obtained by merging two datasets (see Table 1 for a detailed description) collected for different projects (dataset 1 collected between the 4th of October 2012 and the 18th of May 2015, dataset 2 collected between the 18th of January 2019 and the 23rd of February 2022), but obtained from the same MRI scanner. Results from the first MRI dataset and unrelated to anatomical alterations of the hypothalamus have already been published [11, 14, 15]. Notably, the algorithm employed to segment the hypothalamus, as discussed in the introduction, is robust across different datasets [7].

**Table 1:** Demographic and clinical data from the final sample employed for data analyses (2 eCH participants excluded for algorithm failure). The number of patients with ongoing prophylactic treatment also comprises the number of patients with ongoing lithium treatment

				**Statistics and *p*-values**					
	**cCH**	**eCH**	**CTRL**	**cCH vs eCH**		**cCH vs CTRL**		**eCH vs CTRL**	
Participants	57	48	59	n.a		n.a		n.a	
Age (ys; M ± SD)	45 ± 11	46 ± 11	44 ± 10	U = 1243	0.42	U = 1653;	*p* = 0.88	U = 1268	*p* = 0.355
Females/Males	11/46	5/43	12/47	X^2^ (1,105) = 1.59	0.21	X^2^ (1,116) = 0.02	*p* = 0.89	X^2^ (1,107) = 1.95	*p* = 0.163
Left, right, shifting CH attacks	20, 29, 8	22, 22, 4	n.a	X^2^ (1,105) = 0.02	0.88	n.a		n.a	
Daily attacks	3 ± 2	2.5 ± 1.6	n.a	U = 1291	0.19	n.a		n.a	
Years of chronic disease (M ± SD)	8 ± 6.8	n.a	n.a	n.a	n.a	n.a		n.a	
Pts under Lithium	15	4	n.a	X^2^ (1,105) = 5.68	0.017*	n.a		n.a	
Pts under prophylactic treatment	45	17	n.a	X^2^ (1,105) = 20.42	< 0.001*	n.a		n.a	
VAS before MRI (Median- range)	0 (0–3)	0 (0)	n.a	U = NaN (var. = 0)	n.a	n.a		n.a	
	**cCH (DS1)**	**cCH (DS2)**		**Statistics and *p*-values**		**eCH ‘in’**	**eCH ‘out’**	**Statistics and *p*-values**	
Participants	28	29		n.a		23	25		
Age (ys; M ± SD)	45 ± 11	45 ± 10		U = 397	0.89	51 ± 8	42 ± 11	U = 414	0.009*
Females/Males	5/23	6/23		X^2^ (1,57) = 0.073	0.79	1/22	4/21	X^2^ (1, 48) = 1.74	0.19
Left, right, shifting CH attacks	12, 15, 1	8, 14, 7		X^2^ (1,57) = 0.16	0.7	9, 13, 1	13, 9, 3	X^2^ (1, 48) = 2.32	0.15
Daily attacks (M ± SD)	4 ± 2.1	2 ± 1.3		U = 627	< 0.001*	2.5 ± 1.9	2.4 ± 1.2 (**)	U = 181	0.62
Years of chronic disease (M ± SD)	7 ± 6.2	9 ± 7.4		U = 349	0.37	n.a	n.a	n.a	
Pts under Lithium	11	4		X^2^ (1, 57) = 4.77	0.029*	4	0	X^2^ (1, 48) = 4.74	0.029*
Pts under prophylactic treatment	24	21		X^2^ (1, 57) = 1.52	0.22	16	1	X^2^ (1, 48) = 22.5	< 0.001*

*Abbreviations*: *CH* Cluster headache, *cCH* Chronic cluster headache, *eCH* Episodic cluster headache, *DS* Dataset, *n.a.* not applicable, *var.* Variance, *in* in-bout, *out* out-of-bout

(**) when in-bout, * significant value for *p *<0.05

#### Participants

In this study, a total of 107 adult CH patients were enrolled (> 18 years old): 57 had a cCH diagnosis (28 patients were acquired for dataset 1 and 29 for dataset 2) and 50 had a eCH diagnosis (25 eCH ‘in-bout’ phase, 25 in ‘out-of-bout’ phase, all acquired for dataset 2). A control (CTRL) group of 59 self-reported healthy individuals (28 participants acquired for dataset 1 and 31 acquired for dataset 2) with no history of primary headache or chronic pain were also enrolled. The diagnosis of CH was made by senior neurologists (M.L., L.G., and A.P.C.) according to the Diagnostic criteria of the International Classification of Headache (Dataset 1: Headache Classification Committee of the International Headache Society (IHS), 2013 [20]; Dataset 2: The International Classification of Headache Disorders, 3rd Edition, 2018 [21]). Patients with a concomitant diagnosis of other primary or secondary headache disorders, neurological diseases, cardiovascular diseases, diabetes mellitus, or hypertension were excluded from the study, as well as individuals reporting MRI contraindications or identified with abnormal MRI findings.

Two eCH ‘in-bout’ phase participants were excluded for algorithm failure during the segmentation process (see Table 1 for demographical and clinical data) leaving a total of 105 CH patients. The cCH group and the eCH group did not differ from the CTRL groups in terms of age and sex. The level of cranial pain immediately before the MRI session was assessed on a Visual Analog Scale (0 = no pain, 10 = the worst pain imaginable) [10]: both groups had a median of 0 (cCH range 0–3; eCH range: 0–0), and no patient reported being under CH attack during the morphological MRI acquisition.

Notably, the cCH patients of dataset 1 and dataset 2 differed for the number of headache attacks per day (patients from dataset 1: 4 ± 2.1; patients from dataset 2: 2 ± 1.3; U = 627, *p* < 0.001) and for the proportions of participants under lithium treatment [X^2^ (1, 57) = 4.77, *p* = 0.029]. Moreover, the cCH and the eCH ‘in-bout’ groups did not differ in terms of patients under lithium [X^2^(1, 80) = 0.72, *p* = 0.40] and prophylactic treatments [X^2^(1, 80) = 0.80, *p* = 0.37].

The study was planned and conducted in agreement with the latest revision of the Helsinki Declaration and approved by the Ethical Committee of the IRCCS Neurological Institute Carlo Besta. Each participant gave prior written informed consent.

MRI data of both datasets were acquired on the same 3 T scanner (Achieva TX, Philips Healthcare BV, Best, NL) equipped with a 32-channels coil at the Neurological Institute Carlo Besta. All participants underwent a single MRI session, comprising a volumetric high-resolution structural 3D T1-weighted (3D-T1w) image with slightly different parameters (*Dataset 1*: TR = 9.86 ms, TE = 4.59 ms, FOV = 240 × 240 mm, voxel size = 1 mm^3^, flip angle = 8◦, 185 sagittal slices; *Dataset 2*: TR = 8.3 ms, TE = 3.8 ms, FOV = 240 × 240 mm, voxel size = 1 mm^3^, flip angle = 8◦, 180 sagittal slices). For each participant, ‘*recon-all’* algorithm of FreeSurfer software was applied to 3D-T1w images.

Segmentation outputs were visually checked and corrected manually by expert operators (G.D., D.F., G.C.) blinded to the condition of every individual (cCH, eCH, or CTRL) to remove any inaccuracies in the pial-white boundary surfaces segmentation. Then, automated segmentation of hypothalamic subunits was performed employing the ‘*mri_segment_hypothalamic_subunits*’ algorithm of FreeSurfer software v7.2 (https://surfer.nmr.mgh.harvard.edu/fswiki/HypothalamicSubunits)*.*

**Fig. 1:**
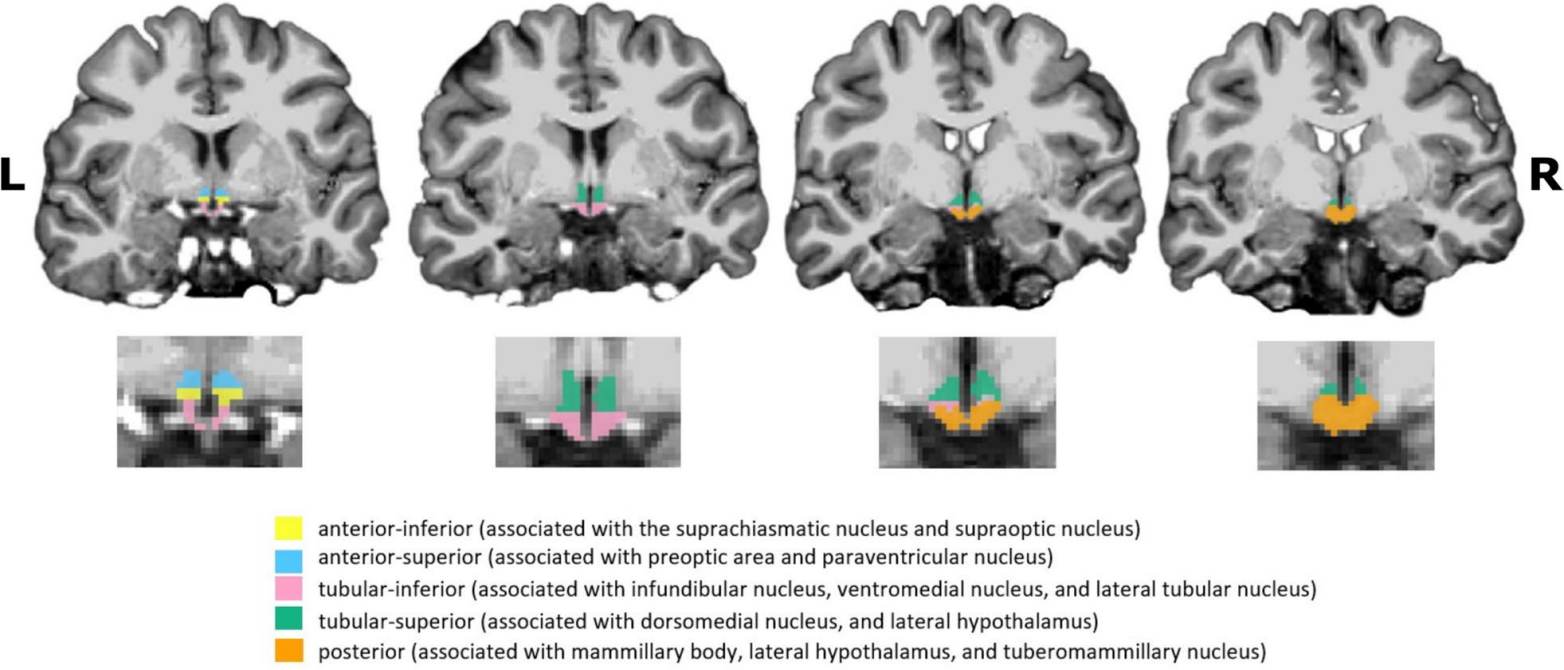
Coronal MRI images (3D T1-weighted) of the segmentation of the hypothalamus (with magnification) of one brain participant from the investigated sample obtained with FreeSurfer software v7.2 (https://surfer.nmr.mgh.harvard.edu/fswiki/HypothalamicSubunits). Abbreviations: R: right, L: left

The automatic algorithm segmented the following bilateral subunits: anterior-inferior (associated with the suprachiasmatic nucleus and supraoptic nucleus), anterior–superior (associated with the preoptic area and paraventricular nucleus), posterior (associated with the mammillary body, lateral hypothalamus, and tuberomammillary nucleus), tubular-inferior (associated with the infundibular nucleus, ventromedial nucleus, and lateral tubular nucleus) and tubular-superior (associated with the dorsomedial nucleus, paraventricular nucleus, and lateral hypothalamus) [7, 35] (Fig. 1). For each participant, the segmentation pipeline produced the mask of each hypothalamic subunit (visually checked by expert operators: G.D., D.F., G.C.) and their respective volumes (expressed in mm^3^. Intracranial volume (ICV of each participant was obtained employing CAT12 (CAT12; [17], http://www.neuro.uni-jena.de/cat/).

#### Statistical analyses

All the statistical analyses were conducted on the sample resulting from the union of dataset 1 and dataset 2 using *JASP* (v. 0.17.1.0) (https://jasp-stats.org). Due to the solid a priori hypothesis of an involvement of the ipsilateral hypothalamus in CH condition, we conducted the analyses separately for the ipsilateral and contralateral-to-the-cranial pain (hereafter, only defined as ‘ipsilateral’ or ‘contralateral’) hypothalamic subunit volumes: the ipsilateral measures correspond to the left side of the brain of CTRL participants while the contralateral measures matched to the right volumes of the brain of CTRL participants. The volumes of the patients suffering from shifting attacks (experiencing right-sided and left-sided attacks) were matched according to the original lateralization of the attacks (i.e., left measures matched to the left volumes of the brain of CTRL participants).

After verifying the presence of no outliers for ICV employing the interquartile range (IQR) method [66], the extracted volumes (VOIs) of the hypothalamic subunits were then corrected for brain size with the *residuals* method [38, 55]. Briefly, for each participant and each VOI, the adjusted volume of interest (*adjVOI*) was computed based on the equation *adj*VOI = VOI—b(ICV—ICV_*mean*_) where the parameter b is the slope of the ICV-VOI regression line, and ICV_*mean*_ is the mean of the ICV values of CTRL group. Notably, we computed the *adjVOIs* separately for dataset 1 and dataset 2 based on the b values and ICV_*mean*_ obtained from the respective CTRL group.

##### Predicting the diagnosis from the hypothalamic subunits

The *adjVOIs* were then entered into binary logistic regression models to identify if they could distinguish patients from CTRL. In particular, we verified their statistical association with respect to (1) the cCH and CTRL diagnosis and (2) the eCH and CTRL diagnosis.

For each statistical association, we used a 3-block model comprising: model #1, including the demographic/clinical variables only (dataset to which the individual belongs -dataset 1 or dataset 2-, age, sex, type of attacks—unilateral or shifting); model #2, including model#1 as null model and the *adjVOIs* of the contralateral hypothalamic subunits; model #3 including model #1 and #2 as null model and the *adjVOIs* of the ipsilateral hypothalamic subunits. The diagnostic discrimination accuracies of the logistic regression models were evaluated using the area under a receiver operating characteristics curve (AUC). Odds ratios and the corresponding *p*-values (computed with the Wald test, testing for the significance of individual coefficients in the model) were also calculated for each hypothalamic subunit. Logistic regression models were used since they do not require a linear relationship between the predictor variable and the response variable and the normal distribution and the constant variance (homoscedasticity) of the residuals. All the results were considered significant for *p* ≤ 0.05.

As the patients with cCH from the two datasets differed in terms of the number of daily headaches, we performed the same analysis as above (3-block logistic regression model) only for the patients with the highest number of attacks (cCH from dataset 1).

##### Identifying possible bias due to lateralization effects

To identify possible bias in the previous analyses due to physiological lateralization effects (i.e., volume asymmetry), we performed a series of t-tests in a hierarchical sequence**.** For this purpose, *adjVOIs* were log-transformed and then checked for normality using Shapiro–Wilk test. When normality was violated, non-parametric t-tests were applied.

First, we tested whether, in the CTRL individuals, there were differences between the left and right subunits that emerged as significant from the logistic regression models (subunits of interest) using one-tail paired-sample t-tests. Using a conservative approach, in the case of significant differences, we considered the results of the logistic regression models as biased by physiological asymmetry and no longer considered them relevant for this work.

Secondly, we checked whether the remaining subunits of interest (i.e., which emerged as significant from the logistic regression models and showing no lateralization effects in the CTRL sample) in the patients’ group (cCH or eCH) also retained their significance compared to the mean of the corresponding right and left subunits in the CTRL group. For this purpose, we performed independent sample t-tests with and without patients with shifting CH attacks. In the case of non-significant values, we cautiously interpreted the results biased by subtle differences between the left and right corresponding subunits in CTRL participants.

Thirdly, we tested whether in the CH group (cCH or eCH) there were significant differences between the homologous (ipsilateral and contralateral) structures of the remaining subunits of interest (i.e., emerged as significant from the logistic regression models, showing no lateralization effects in the CTRL sample, and maintaining their significant effects also when compared to the mean values of the left and right subunit of CTRL participants). For this purpose, we used the one-tail paired-sample t-test. Again, we performed the analyses with and without patients with shifting CH attacks. All the results were considered significant for *p* ≤ 0.05.

##### Effects of years of chronic disease and lithium therapy in cCH patients

Correlation analyses were used to identify whether, in cCH patients, the volumes (expressed as log-transformed *adjVOIs*) of the subunits of interest (i.e., emerged as significant from the logistic regression models, showing no lateralization effects in the CTRL sample and maintaining their significant effects also when compared to the mean values of the left and right subunit of CTRL participants) correlated with years of the chronic condition or with the numbers of headache attacks per day.

Moreover, a binary logistic regression model was used to identify whether the volumes of the subunits of interest (predictors expressed as *adjVOIs*) were associated with the ongoing Lithium therapy (dependent variable). To this aim, we used a 2-block model with model #1 including the demographic/clinical variables only (dataset, age, sex, type of attacks) and model #2, including model #1 as null model and the *adjVOIs* of the subunits of interest. All the results were considered significant for *p* ≤ 0.05.

### Functional connectivity of hypothalamic subunits in healthy participants

As a post-hoc approach, to determine whether the hypothalamic subunits identified as abnormal in CH patients presented robust functional connectivity with areas of the mesocorticolimbic system under physiological conditions rs-fMRI data from 167 participants (age: M = 29.3, SD = 3.3; 99 females) of the publicly available Human Connectome Project dataset (HCP—Young Adult; for details https://www.humanconnectome.org/hcp-protocols-ya-7t-imaging [56, 64]) were used. To this end, a region of interest-to-region of interest (ROI-to-ROI) connectivity analysis with CONN toolbox v21a (www.nitrc.org/projects/conn) [49], was performed. As declared by HCP, all participants provided written informed consent to the study and the sharing of de-identified data.

For each participant, we employed the 4 rs-fMRI data runs acquired at 7 T (900 volumes per run, 1.6 mm isotropic voxels, TR = 1000 ms, TE = 22.2 ms, flip angle = 45 degrees, FOV = 208 × 208 mm; [56, 63] and already preprocessed (HCP filename: ‘*rfMRI ∗ hp2000_clean.nii.gz’*). The HCP rs-fMRI preprocessing steps comprised gradient nonlinearity-induced distortion correction, rigid body head motion correction, EPI image distortion correction, co-registration between the fMRI and structural data, normalization to MNI space, high-pass filtering, brain masking [18] and independent components analysis-based artifact removal of noise components [53]. As structural MRI data, we employed, as indicated by HCP for the 7 T rs-fMRI dataset, the 3 T T1-weighted image resampled at 1.6 mm resolution (HCP filename: ‘*T1w_restore.1.6.nii.gz’*).

To produce reliable functional connectivity results, we defined the anatomical ROIs of the hypothalamic subunits at the single-subject level by applying the ‘*mri_segment_hypothalamic_subunits’* algorithm (FreeSurfer v7.2) to the 3 T T1-weighted image at 1 mm resolution (as recommended in https://surfer.nmr.mgh.harvard.edu/fswiki/HypothalamicSubunit—HCP filename: ‘*T1w_restore.nii.gz’*) of each participant. From the initial dataset, one participant was excluded due to an error in the segmentation process. The following ROIs for the mesocorticolimbic system were selected: the nucleus accumbens, the amygdala, the hippocampus, the medial and orbital prefrontal cortex, and the frontal pole from the Harvard–Oxford atlas available in CONN, while the ROI of the ventral tegmental area from a publicly available probabilistic atlas [61]. The subject-specific ROIs of the hypothalamic subunits emerged as significant from the logistic regression models, as well as mesocorticolimbic ROIs were used in the ROI-ROI analysis in CONN. Then, MRI data underwent the following denoising steps: identification of outlier volumes through Artifact Detection Tools (ART), functional smoothing (FWHM = 6 mm), and physiological denoising through *aCompCor*. Subsequently, in each participant, the mean average BOLD time series of each selected ROI (extracted from the unsmoothed and denoised rs-fMRI volumes) was computed, and for each possible pair of ROIs, a Fisher transformed bivariate correlation coefficient was obtained. ROI-to-ROI rs-fMRI functional connectivity matrices were calculated, and parametric multivariate statistics were applied (cluster threshold: *p* < 0.05 cluster-level, p-FDR corrected—MVPA omnibus test; connection threshold: *p* < 0.05 uncorrected).

## Results

### Predicting the diagnosis from the hypothalamic subunits

**Fig. 2:**
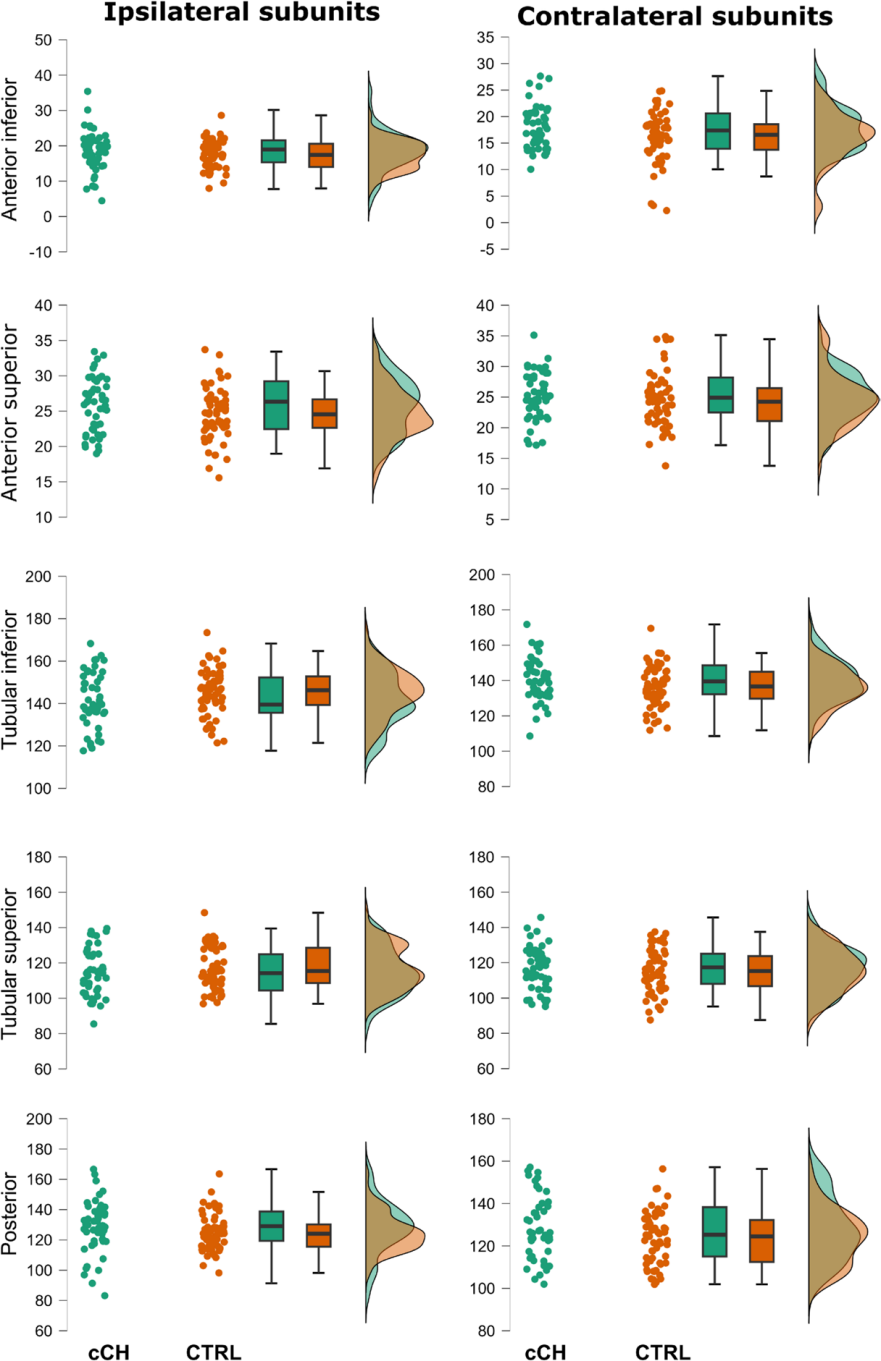
Plots of the volumes of the hypothalamic subunits normalized for the brain size according to the *residual method* (adjVOIs) in cCH and CTRL participants. For representational purposes, patients with shifting attacks were excluded. Abbreviations: cCH patients: chronic cluster headache patients, CTRL: control participants

**Fig. 3:**
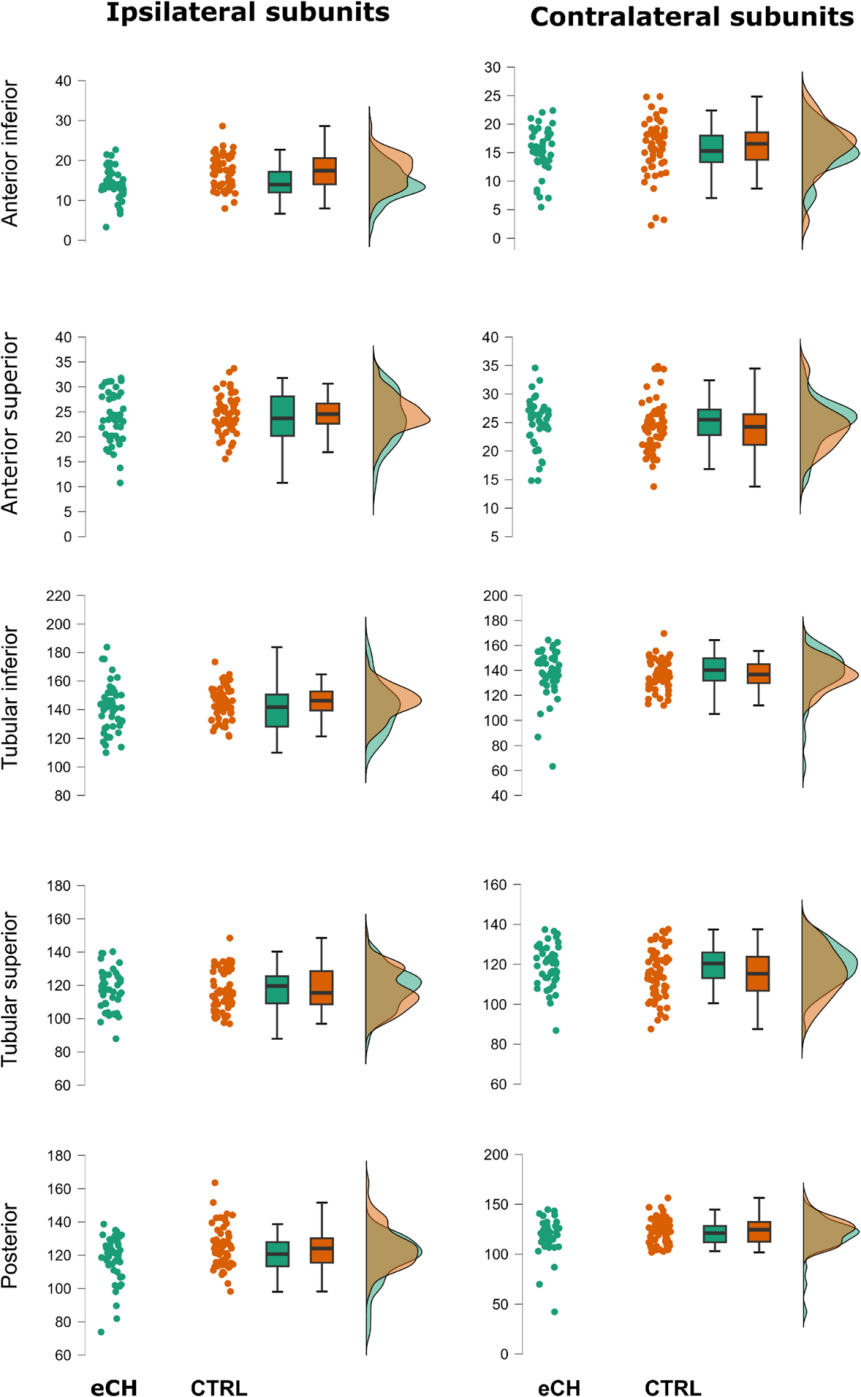
Plots of the volumes of the hypothalamic subunits normalized for the brain size according to the *residual method* (adjVOIs) in eCH and CTRL participants. For representational purposes, patients with shifting attacks were excluded. Abbreviations: eCH: episodic cluster headache patients, CTRL: control participants

Descriptive statistics for the *adjVOI* of the hypothalamus subunits are reported in Table 2 (see also Figs. 2 and 3). Logistic regression results are reported in Tables 3 and 4. In the 3-block logistic regression model, the diagnosis of cCH with respect to CTRL was significantly better predicted by model #3 [i.e., demographic/clinical variables and contralateral hypothalamic subunits as null model and ipsilateral hypothalamic subunits (Χ^2^(101) = 11.74, *p* = 0.039, Nagelkerke *R*^2^ = 0.137)] than by model #2 (i.e., demographic/clinical variables ad null model and contralateral hypothalamic subunits), which, on the other hand, did not yield significant results (Χ^2^(106) = 8.044, *p* = 0.154, Nagelkerke *R*^2^ = 0.093) compared to model #1 [i.e., demographic/clinical variables; (Χ^2^(111) = 12.67, *p* = 0.013, Nagelkerke *R*^2^ = 0.138)]. As expected, model #3 achieved better performance (AUC = 0.77) in comparison to model #2 (AUC = 0.69) and model #1 (AUC = 0.61). These results indicate that only the ipsilateral, and not the contralateral, subunits could discriminate cCH from CTRL participants. Importantly, among the ipsilateral measures, only the anterior–superior and tubular-inferior subunit significantly predicted the diagnosis (respectively, OR = 1.16, *p* = 0.034; OR = 0.95, *p* = 0.037). More specifically, the ipsilateral anterior–superior subunit presented a larger volume in cCH patients (M = 25.62 mm^3^; 95% CI = 24.6–26.6 mm^3^) in comparison to CTRL participants (M = 24.51 mm^3^; 95% CI = 23.57–25.47), while tubular-inferior subunit presented an opposite pattern (respectively, M = 142.18 mm^3^; 95% CI = 138.73–145.64 in cCH patients and M = 145.4 mm^3^; 95% CI = 142.60- 148.20 in CTRL individuals).

**Table 2:** Descriptive statistics for the volumes of each subunit corrected in respect to the total intracranial volume (computed with CAT12) employing the *residuals method* (*adjVOI*, see the main text)

	**Ipsilateral Subunits Volumes (mm**^**3**^**)**					**Contralateral Subunits Volumes (mm**^**3**^**)**				
**cCH patients**	**Ant. inf**	**Ant. sup**	**Posterior**	**Tub. inf**	**Tub. sup**	**Ant. inf**	**Ant. sup**	**Posterior**	**Tub. inf**	**Tub. sup**
Median	18.32	25.91	128.56	139.52	114.11	17.26	24.81	125.29	139.23	116.13
Mean	18.25	25.62	128.05	142.18	114.29	17.47	24.69	127.20	139.56	115.64
95% CI Mean Upper	19.64	26.60	132.47	145.64	117.59	18.54	25.75	131.16	142.79	118.80
95% CI Mean Lower	16.85	24.64	123.63	138.73	110.99	16.40	23.63	123.25	136.32	112.48
Maximum	4.46	18.98	83.19	117.70	85.47	8.56	17.15	97.36	108.60	95.21
Minimum	35.41	33.42	166.63	180.53	139.51	27.63	35.11	157.14	171.78	145.73
**eCH patients**										
Median	13.98	23.41	120.67	141.66	118.42	15.30	25.20	121.64	138.17	119.73
Mean	14.32	23.60	118.05	141.19	117.36	14.83	24.48	118.36	137.14	118.48
95% CI Mean Upper	15.46	25.08	121.94	145.86	120.74	16.06	25.72	123.49	142.45	121.64
95% CI Mean Lower	13.18	22.13	114.15	136.52	113.98	13.61	23.24	113.24	131.83	115.32
Maximum	3.31	10.77	73.83	110.02	87.88	5.17	14.82	42.23	63.35	86.83
Minimum	22.67	31.78	138.68	183.76	140.22	22.38	34.58	144.71	164.29	137.38
**CTRL participants**										
Median	17.45	24.55	124.05	146.26	115.44	16.56	24.24	124.48	136.64	115.27
Mean	17.29	24.52	124.70	145.40	117.07	16.11	24.41	123.31	136.53	115.40
95% CI Mean Upper	18.33	25.47	127.84	148.20	120.13	17.32	25.53	126.49	139.50	118.53
95% CI Mean Lower	16.25	23.57	121.55	142.60	114.00	14.91	23.29	120.12	133.56	112.26
Maximum	7.97	15.57	98.21	121.43	96.88	2.27	13.77	101.89	111.89	87.58
Minimum	28.61	33.70	163.56	173.40	148.43	24.84	34.87	156.34	169.52	137.54

**Table 3:** 3-block binary logistic regression models (diagnosis as response variable) results comprising: model #1, including the demographic/clinical variables only (dataset to which the individual belongs -dataset 1 or dataset 2-, age, sex, type of attacks—unilateral or shifting); model #2, including model#1 as null model and the *adjVOIs* of the contralateral hypothalamic subunits; model #3 including model #1 and #2 as null model and the *adjVOIs* of the ipsilateral hypothalamic subunits

Model	df	Χ^2^	*p*	*R*^2^	AUC	Sensitivity	Specificity	Precision
**cCH vs. CTRL**								
Intercept	115							
Model 1 (DEM)	111	12.669	**0.013**	0.138	0.609	0.439	1	1
Model 2 (DEM + CONTRA)	106	8.044	0.154	0.093	0.693	0.579	0.729	0.673
Model 3 (DEM + CONTRA + IPSI)	101	11.737	**0.039**	0.137	0.767	0.667	0.78	0.745
**eCH vs. CTRL**								
Intercept	106							
Model 1 (DEM)	102	47.852	**< .001**	0.483	0.814	0.896	0.593	0.642
Model 2 (DEM + CONTRA)	97	10.054	0.074	0.148	0.875	0.813	0.78	0.75
Model 3 (DEM + CONTRA + IPSI)	92	22.796	**< .001**	0.339	0.936	0.833	0.847	0.816

*Abbreviations*: *cCH* Chronic cluster headache, *eCH* Episodic cluster headache, *CTRL* Healthy participants, *AUC* Area under a receiver operating characteristics curve, *DEM* Demographic/clinical variables, *IPSI* Ipsilateral-to-the cranial pain subunits, *CONTRA* Contralateral to the cranial pain subunits

**Table 4:** Parameters of the full models (model #3) of the 3-block binary logistic regression models (diagnosis as response variable) comprising including model #1 and #2 as null model. *P*-values were computed with the Wald test testing for the significance of individual coefficients in the model

Parameters	b	SE	OR	z	p	LB	UB
	**cCH vs CTRL**						
Intercept	-4.498	4.221	0.011	-1.066	0.287	-12.770	3.775
Sex	0.094	0.562	1.098	0.167	0.867	-1.007	1.195
Age	0.015	0.024	1.016	0.653	0.514	-0.031	0.062
Type of attacks	18.588	1266.085	1.183 × 10^+8^	0.015	0.988	-2462.892	2500.069
Dataset	-0.107	0.544	0.898	-0.197	0.843	-1.174	0.959
CONTRA Anterior-Inferior	0.032	0.069	1.032	0.458	0.647	-0.104	0.167
CONTRA Anterior–Superior	-0.015	0.066	0.985	-0.230	0.818	-0.145	0.115
CONTRA Posterior	0.007	0.026	1.007	0.281	0.778	-0.043	0.058
CONTRA Tubular-Inferior	0.052	0.024	1.053	2.168	0.030	0.005	0.099
CONTRA Tubular-Superior	0.001	0.026	1.001	0.022	0.982	-0.051	0.052
IPSI Anterior-Inferior	0.016	0.063	1.016	0.255	0.799	-0.107	0.139
IPSI Anterior–Superior	0.147	0.070	1.159	2.117	0.034	0.011	0.284
IPSI Posterior	0.016	0.020	1.016	0.789	0.430	-0.023	0.055
IPSI Tubular-Inferior	-0.050	0.024	0.951	-2.084	0.037	-0.097	-0.003
IPSI Tubular-Superior	-0.030	0.025	0.970	-1.220	0.222	-0.079	0.018
	**eCH vs CTRL**						
Intercept	-50.762	3408.109	8.998 × 10^–23^	-0.015	0.988	-6730.533	6629.008
Sex	0.353	0.800	1.423	0.441	0.659	-1.215	1.921
Age	0.067	0.035	1.069	1.890	0.059	-0.002	0.136
Type of attacks	20.416	4614.617	7.355 × 10^+8^	0.004	0.996	-9024.068	9064.900
Dataset	22.384	1704.051	5.261 × 10^+9^	0.013	0.990	-3317.495	3362.262
CONTRA Anterior-Inferior	0.127	0.093	1.135	1.358	0.174	-0.056	0.310
CONTRA Anterior–Superior	0.157	0.114	1.170	1.381	0.167	-0.066	0.380
CONTRA Posterior	0.062	0.041	1.064	1.506	0.132	-0.019	0.143
CONTRA Tubular-Inferior	-0.039	0.032	0.962	-1.225	0.221	-0.101	0.023
CONTRA Tubular-Superior	0.070	0.043	1.073	1.629	0.103	-0.014	0.155
IPSI Anterior-Inferior	-0.279	0.124	0.756	-2.243	0.025	-0.523	-0.035
IPSI Anterior–Superior	-0.222	0.111	0.801	-2.006	0.045	-0.440	-0.005
IPSI Posterior	-0.006	0.044	0.994	-0.127	0.899	-0.092	0.081
IPSI Tubular-Inferior	-0.068	0.030	0.934	-2.299	0.022	-0.126	-0.010
IPSI Tubular-Superior	0.065	0.043	1.067	1.495	0.135	-0.020	0.149

*Abbreviations*: *cCH* Chronic cluster headache, *eCH* Episodic cluster headache, *CTRL* Healthy participants, *IPSI* Ipsilateral-to-the cranial pain subunits, *CONTRA* Contralateral to the cranial pain subunits

The 3-block binary logistic regression model perfomed only on cCH patients of dataset 1 and CTRL participants showed that the diagnosis of cCH was significantly better predicted by model #3 [i.e., demographic/clinical variables and contralateral hypothalamic subunits as null model and ipsilateral hypothalamic subunits (Χ^2^(42) = 29.1, *p* < 0.001, Nagelkerke *R*^2^ = 0.556)] than by model #2 (i.e., demographic/clinical variables as null model and contralateral hypothalamic subunits), which, on the other hand, did not yield significant results (Χ^2^(47) = 7.029, *p* = 0.219, Nagelkerke *R*^2^ = 0.159) compared to model #1 (i.e., demographic/clinical variables). As expected, model #3 achieved better performance (AUC = 0.90) in comparison to model #2 (AUC = 0.70) and model #1 (AUC = 0.53). In this case, the only significant hypothalamic subunit in the full model was the ipsilateral anterior–superior subunit (OR = 1.87, *p* = 0.001).

Also with regard to eCH patients, the 3-block model showed that diagnosis of eCH with respect to CTRL was significantly better predicted by model #3 [i.e., demographic/clinical variables, contralateral hypothalamic subunits and ipsilateral hypothalamic subunits (Χ^2^ (92) = 22.796, *p* < 0.001, Nagelkerke *R*^2^ = 0.339) compared to model #2 (i.e., demographic/clinical variables and contralateral hypothalamic subunits) which did not yield significant results (Χ^2^(97) = 10.05, *p* = 0.074, Nagelkerke *R*^2^ = 0.148) compared to model #1 [i.e., demographic/clinical variables; (Χ^2^(102) = 47.85, *p* < 0.001, Nagelkerke *R*^2^ = 0.483)]. In agreement, model #3 performed better (AUC = 0.94) than model #2 (AUC = 0.88) and model #1 (AUC = 0.81). Also in this case, the results indicate that only the ipsilateral, and not the contralateral, subunits could discriminate eCH from CTRL participants. Remarkably, among the ipsilateral measures, the anterior-inferior (OR = 0.756, *p* = 0.025), anterior–superior (OR = 0.801, *p* = 0.045), and tubular-inferior subunit (OR = 0.934, *p* = 0.022) significantly predicted the diagnosis. More specifically, all these subunits presented a smaller medium volume in eCH patients (anterior-inferior: M = 14.32 mm^3^, 95% CI = 13.18–15.46; anterior–superior: M: 23.60 mm^3^, 95% CI = 22.13–25.8; tubular-inferior: M = 141.19 mm^3^, 95% CI = 136.52–145.86) in comparison to CTRL participants (anterior-inferior: M = 17.29 mm^3^, 95% CI = 16.25–18.33; anterior–superior: M = 24.52 mm^3^, 95% CI = 23.57–25.47; tubular-inferior: M = 145.40 mm^3^, 95% CI = 142.60–148.22).

### Identifying possible bias due to lateralization effects

The anterior-inferior, anterior–superior, and tubular-inferior subunits emerged as significant from the logistic regression models were considered.

The one-tail paired-sample t-test investigating the possible lateralization effects in CTRL participants for the subunits of interest (i.e., that were shown to discriminate cCH from CTRL and eCH from CTRL) indicated that the left anterior-inferior subunit and tubular-inferior subunit were significantly different from their right counterparts (respectively, W = 1145, z = 1.962, *p* = 0.025; T(58) = 7.139, z = 1.962, *p* < 0.001). No difference was instead detected between the left and right anterior–superior subunits (T(58) = 0.340, *p* = 0.367). Thus, subsequent analyses were only applied to test abnormalities in the ipsilateral anterior–superior subunit in both CH groups (i.e., cCH and eCH).

The one-tail independent sample t-tests investigating the differences between the ipsilateral anterior–superior subunit of cCH patients and the mean of the left and right respective subunit in CTRL participants showed that this subunit was significantly different between the two groups either when computed with patients suffering from shifting attacks (T(114) = 1664, *p* = 0.049) and without (T(106) = 1.993,*p* = 0.024). Notably, we did not find a similar effect for eCH patients (with patients with shifting attacks: W = 1281, *p* = 0.200; without patients with shifting attacks: W = 1191, *p* = 0.239). Thus, as a precautionary measure, in eCH patients, we did not consider anterior–superior subunit.

The one-tail paired-sample t-test investigating the differences between the ipsilateral and contralateral anterior–superior subunits in cCH patients showed that these two subunits were significantly different (T(56) = 1.706, *p* = 0.047). However, when patients with shifting attacks (T(48) = 1.331, *p* = 0.095) were excluded, the effect did not reach the significance. Notably, ipsilateral subunit presented larger volumes in comparison to the contralateral subunit with (ipsilateral: M = 25.62, 95% CI = 24.64–26.60, contralateral: M = 24.69, 95% CI = 23.63–25.75) and without (ipsilateral: M = 25.91, 95% CI = 24.83–26.99 contralateral: M = 25.09, 95% CI = 24.00–26.19) patients with shifting attacks. We computed the coefficients of variation, which showed more variability for the contralateral subunits (sample with patients with shifting attacks: 0.053, sample without patients with shifting attacks: 0.050) with respect to the ipsilateral subunits (sample with patients with shifting attacks: 0.046, sample without patients with shifting attacks: 0.047).

### Effects of years of chronicisation and Lithium therapy in cCH patients

**Fig. 4:**
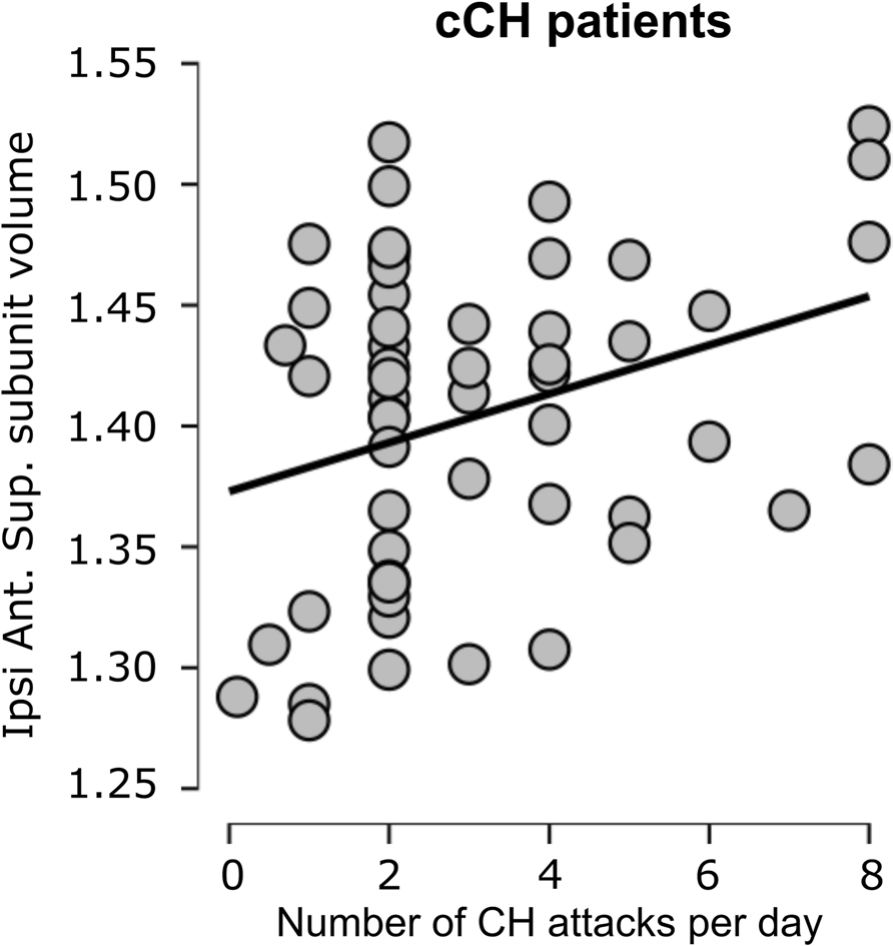
Scatter plot of the correlation between the volumes of the ipsilateral antero-superior hypothalamic subunit and the number of attacks per day in cCH patients (chronic cluster headache patients). Volumes are expressed as LOG of the normalized measures (*adjVOI*)

In cCH patients, we found a significant linear association between the anterior–superior subunit volumes and the daily number of cluster headache attacks (*r* = 0.311, *p* = 0.020 – normality data distribution checked with Shapiro–Wilk) (see Fig. 4), while no significant linear association was observed with the years of chronic disease (*r* = -0.039, *p* = 0.773, – normality data distribution checked with Shapiro–Wilk).

Furthermore, in the 2-block logistic regression model investigating the role of lithium therapy in determining the observed hypothalamic abnormalities, model #2 did not better discriminate between cCH patients on the basis of lithium therapy (Χ^2^(51) = 3.32, *p* = 0.068, Nagelkerke *R*^2^ = 0.091) with respect to model #1 [i.e., demographic/clinical variables] (Χ^2^(52) = 9.75, *p* = 0.045, Nagelkerke *R*^2^ = 0.230).

### Functional connectivity of hypothalamic subunits of interest

**Fig. 5:**
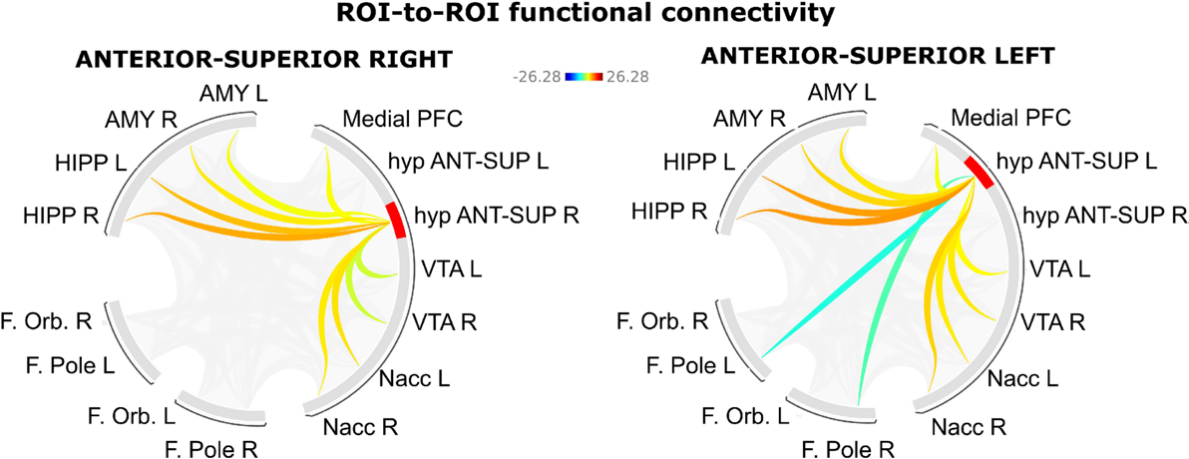
ROI-to-ROI functional connectivity from 166 healthy individuals of the 7 T Human Connectome Project (HCP) rs-fMRI dataset for the anterior–superior hypothalamic sub-unit within the areas/structures of the mesocorticolimbic system. Results are significant for parametric multivariate statistics (cluster threshold: *p* < 0.05 cluster-level, *p*-FDR corrected—MVPA omnibus test; connection threshold: *p* < 0.05 uncorrected). Abbreviations: hyp ANT-SUP = anterior–superior hypothalamic sub-unit, Medial PFC = medial prefrontal cortex, AMY = amygdala, HIPP = hippocampus, F.Orb. = frontal orbital, F Pole = frontal pole, VTA = ventral tegmental area, Nacc = nucleus accumbens, R = right, L = left

Results of rs-fMRI data from the HCP dataset in healthy participants showed that both right and left antero-superior hypothalamic subunits exhibit robust functional connectivity extending bilaterally with all subcortical structures of the mesocorticolimbic system (nucleus accumbens, amygdala, hippocampus, bilateral ventral tegmental area) and with the medial frontal cortex (see Additional file 1: Table 1 in SM and Fig. 5).

## Discussion

In this study, we ought to determine the specific hypothalamic nuclei engaged in CH pathophysiology. To this aim, we utilized a state-of-the-art, fully automated algorithm [7] to extract the volumes of hypothalamic subunits (in agreement with the anatomical subdivision proposed by Makris et al. [35] from MRI brain data obtained from a large group of CH patients and from a CTRL group and we performed the relevant comparisons of the obtained normalized measures.

Our results revealed distinct patterns of volumetric differences: specifically, patients with the chronic form of CH (i.e., cCH) exhibited increased volumes of the ipsilateral antero-superior subunit, while no significant evidence of hypothalamic abnormalities was observed in patients with eCH. More importantly, we found that the volumes of this hypothalamic subunit in cCH patients correlate with the number of daily headache attacks, but not with the number of years of chronic disease; moreover, it does not appear to be related to lithium treatment.

Together, these results indicate that the ipsilateral volumetric increase of this region is a biological marker of the chronic form of CH and not a marker of disease progression or Lithium effects.

Our findings not only corroborate previous research highlighting the involvement of the ipsilateral-to-pain hypothalamus in the pathophysiology of CH [33, 45] but also support the recent animal literature indicating that the paraventricular nucleus of the hypothalamus (PVN), located with the preoptic area in the antero-superior subunit, is robustly involved in headache mechanisms [52, 68].

Furthermore, with a post-hoc approach, based on the evidence of close interactions between the PVN and the mesocorticolimbic system [5, 12, 24] and considering the abnormalities of the mesocorticolimbic system observed in cCH patients [14], we used the 7 T MRI rs-fMRI dataset from the Human Connectome Project [56, 64] to investigate the functional connectivity between the anterior–superior hypothalamic subunit and key structures/areas of the mesocorticolimbic system. The high spatial resolution and the high statistical power (4 rs-fMRI runs, each one of 900 volumes, from 166 participants) of this dataset associated with the single-subject level segmentation of the hypothalamic anterior–superior subunit of interest, allowed a definition of the functional connectivity of this area in physiological conditions. The results demonstrate robust functional connectivity between this hypothalamic subunit and the subcortical structures of the mesocorticolimbic system (ventral tegmental area, nucleus accumbens, amygdala, and hippocampus).

The previous literature [45] and the specific clinical features of CH (i.e., unilateral craniofacial pain and ipsilateral-to-the-craniofacial pain trigeminal autonomic symptoms) robustly support an involvement of the ipsilateral hypothalamus in CH. Nevertheless previous neuroimaging studies conducted with VBM reported inconsistent results [2, 41, 48, 69], probably due to the limitations of the applied algorithm [71]. More recently, Arkink et al. [4], employing T1-weighted images acquired with 1.5 T MRI, reported an increase in the volumes of the ipsilateral anterior regions of the hypothalamus (according to the Authors possibly involving the suprachiasmatic nucleus and the PVN) in patients with cCH when employing VBM. However, hypothalamic manual segmentation in the same study revealed an increase in volumes bilaterally in the same region for all CH patients (both chronic and episodic), thus suggesting that manual segmentation may be more sensitive in detecting hypothalamic alterations compared to VBM. It is important to note that although manual segmentation of brain structures on MRI images is considered the gold standard in terms of accuracy, it is prone to significant intra- and inter-observer variability, which can lead to differences in reported results [58]. This variability is particularly challenging for hypothalamic subunits due to their small volumes and lack of MRI contrast, making their manual delineation less reproducible and more prone to variability [7]. Despite the important findings of Arkink et al. [4] indicating alterations in the anterior regions of the hypothalamus, the limitations of manual segmentation could explain the fact that anterior hypothalamus abnormalities were reported bilaterally, a finding that appears to be at odds with the unilateral clinical features of CH.

Our study, using an automated algorithm for hypothalamic subunit segmentation, partially aligns with the findings of Arkink et al. [4] by showing an enlargement of ipsilateral anterior region of the hypothalamus, specifically the ipsilateral anterior–superior hypothalamic subunit, in patients with cCH. Still, it does not replicate the observations of abnormal bilateral anterior hypothalamus in cCH and eCH patients. Interestingly, a recent study by Lee et al. [31] which employed the same algorithm as our study, found no evidence of abnormal hypothalamic subunits in patients with eCH, supporting our findings.

Remarkably, our results are a fundamental step further in the comprehension of the pathophysiology of chronic CH in relation to the possible involvement of PVN [68]. Organized in several discrete subnuclei, the PVN houses the so-called preautonomic neurons of both sympathetic and parasympathetic systems [6] as well as neurons constituting the hypothalamic–pituitary–adrenal (HPA) axis (corticotrophin-releasing hormone (CRH) expressing neurons, projecting to the anterior pituitary where they induce the secretion of adrenocorticotropin hormone (ACTH)) and the hypothalamo-neurohypophyseal system (oxytocin and vasopressin expressing magnocellular neurons projecting to the posterior pituitary where they release oxytocin and vasopressin into the blood circulation) [59].

The PVN hypothalamic nucleus is integral to maintaining autonomic and endocrine homeostasis [26] and it plays a crucial role in orchestrating responses to real or perceived stress by activating, through the CRH, the HPA axis [13]. At this regard, it is well known that CH patients are characterized by HPA hyperactivity [32] and that vagal nerve stimulation, able to control episodic cluster headache attacks when used non-invasively [44] induces anti-inflammatory responses by modulating the activity of the CRH-PVN neurons and thus the HPA axis [8].

Notably, the PVN, along with the supraoptic nucleus, is the only site of oxytocin production in the brain [59]. Oxytocin is a neuropeptide with various physiological actions, including the induction of uterine muscle contractions during childbirth and lactation, as well as the modulation of social behavior, memory, mood, and anxiety [27]. Oxytocin has also been found to play a prominent role in pain modulation through both central and peripheral pathways [25]. Notably, the oxytocin PVN neurons project to the superior salivary nucleus, which is involved in autonomic phenomena of CH attacks [52, 68] and to the caudal spinal trigeminal nucleus (Sp5C) [52]. Recent studies have shown that oxytocin modulates activity in the trigeminal-cervical complex induced by meningeal electrical stimulation and that oxytocin receptors are widely represented in the trigeminovascular system [67]. Importantly, administration of oxytocin has been shown to improve pain in migraine [30, 62].

Moreover, oxytocin PVN projections robustly target the mesocorticolimbic system, an abnormal network in chronic CH patients [14]. Animal studies have indeed demonstrated strong interactions between oxytocin and mesolimbic areas such as the amygdala, nucleus accumbens, and ventral tegmental area [12, 24, 29, 46]. In humans, oxytocin signaling genes are highly co-expressed with several dopaminergic genes, suggesting fundamental gene pathway interactions between oxytocin and the dopaminergic system [51].

Our findings of robust functional connectivity between the anterior–superior hypothalamic subunit and mesocorticolimbic structures (amygdala, hippocampus, nucleus accumbens, ventral tegmental area, and medial prefrontal cortex) in a large independent dataset of healthy participants (HCP 7 T MRI dataset) corroborate this notion and suggest, together with the our previous observation of anatomical and functional alterations in this circuit in patients with cCH [14], that the PVN-mesocorticolimbic route could play a role in the chronic form of CH. Future studies should ascertain this possibility. Remarkably, the utilization of MRI data from healthy participants to infer mechanisms and to enhance the comprehension of neuropathological conditions is not novel. For instance, a seminal study [36] showed that atrophy progression in primary progressive aphasia spread from a central area and extended to other regions, based on the connectivity of this central area observed in healthy control participants. This robustly underscores that studying healthy participants can enhance comprehension of pathological conditions.

Notably, the possible involvement of the preoptic area could explain other main clinical features of CH, such as the cyclic recurrence of craniofacial pain, the nocturnal recurrence and the restless and aggressive behavior during attacks [60], the neuroendocrine abnormalities, and the sleep disturbances not related to nocturnal attacks [34].

Our study has several strengths that contribute to its robustness and reliability. First, we conducted our study on the largest MRI dataset of CH patients investigated so far (57 patients with cCH and 48 patients with eCH). Second, we utilized a fully automated state-of-the-art algorithm for segmenting the hypothalamic subunits, which has been extensively validated and shown to outperform other segmentation methods. This algorithm exploits a deep convolutional neural network, and it was shown to be superior to previous ones for unraveling hypothalamic structural abnormalities, outperforming multi-atlas segmentation approaches and human inter-rater accuracy level, and reaching intra-rater precision [7]. Specifically, this algorithm was trained on 37 T1-weighted brain MRI images that were manually segmented employing the protocol by Makris et al. [35], which, considering the small size of the hypothalamic nuclei, uses only visible anatomical landmarks grouping the hypothalamic nuclei in 5 subunits. To validate their method, Billot et al. [7] showed that their tool maintained high accuracy performances on a low-quality MRI dataset and on a subset of 675 heterogeneous multi-site brain scans (from ADNI). Finally, the authors could replicate the neuropathological atrophy of the hypothalamic subunits associated with Alzheimer’s disease on 317 ADNI scans. This fully automated algorithm [7] thus promises precise quantifications of the hypothalamic subunits. Third, with reference to the optimal solution to correct raw volumes of the structures of interest for head size, we employed the *residuals* method [55], that can robustly control the effects of sex [38, 55]. Furthermore, in addition to reducing the possible residual confounding effects of age and sex by matching groups for these variables, we checked their effects by using them in null models in logistic regression analyses. Fourth, we used binary logistic regression models, which are robust to violations of normal distribution and homoscedasticity assumptions. Fifth, we conducted a thorough assessment of possible bias due to lateralization effects. This comprehensive evaluation allowed us to exclude subunits as possible sites of genuine abnormality and provided greater confidence in our results.

Some limitations of our study, however, should be noted. First, our findings showed that in patients with cCH, the ipsilateral antero-superior subunit was significantly larger than the respective contralateral subunit only when calculated over the entire sample (i.e., including patients with shifting attacks). However, the mean volume of the antero-superior ipsilateral subunit was always larger than the mean of the contralateral subunit also when excluding patients with shifting attacks. Second, although we have controlled the likelihood that lithium may be behind the observed increase in the volume of the abnormal subunit, the effect of other drugs commonly taken by patients with CH (such as antidepressants, corticosteroids, and calcio-antagonists) cannot be ruled out with certainty. However, studies investigating brain changes induced by corticosteroids and antidepressants do not show any effects at hypothalamic level [3, 9, 23]. Moreover, eCH patients in-bout phase (who do not present abnormalities of the anterior superior hypothalamic subunit) and cCH patients did not differ for the proportions of patients employing prophylactic treatments suggesting that the observed effect (ipsilateral anterior–superior subunit enlargement) may indeed be unrelated to drug treatments. Third, lacking a strong a priori hypothesis regarding the engagement of specific hypothalamic nuclei in CH, we did not gather clinical data specifically targeting the anterior–superior hypothalamic subunit.

## Conclusions

We showed that CH in the chronic form is characterized by an abnormal increase in the volume of the ipsilateral-to-the-pain anterior–superior hypothalamic subunit, where PVN and the preoptic area are located. Although our study could not ascertain which of the two nuclei (i.e., PVN, preoptic area) is altered, converging animal studies [16, 52, 67] and clinical evidences [32, 68] but also the results of Arkink et al. [4] support the hypothesis that the PVN could carry out a fundamental role in the pathophysiology of CH.

The divergent results for the cCH and eCH patients (showing no hypothalamic subunit volumes abnormality) indicate that the enlargement of the ipsilateral anterior–superior hypothalamic subunit is linked to the chronic form of CH. Moreover, the evidence that these volumes in patients with cCH are not correlated with the duration of disease chronification or influenced by lithium treatment suggests that the observed volumetric difference may represent a biological trait marker of cCH patients rather than a marker of disease progression or Lithium effects. This is robustly supported by the correlation between the volume of the identified region and the number of headache attacks per days.

### Supplementary Information

**Additional file 1: Table 1SM. **ROI-to-ROI functional connectivity from 166 healthy individuals of the 7T Human Connectome Project (HCP) rs-fMRI dataset for the anterior-superior hypothalamic sub-unit within the areas/structures of the mesocorticolimbic system. Results are significant for parametric multivariate statistics (cluster threshold: *p*<0.05 cluster-level, p-FDR corrected - MVPA omnibus test; connection threshold: *p* < 0.05 uncorrected). Abbreviations: hyp ANT-SUP = anterior-superior hypothalamic sub-unit, Medial PFC= medial prefrontal cortex, VTA = ventral tegmental area, R = right, L = left.

## Data Availability

The Human Connectome Project 7 T rs-fMRI data used in this study is publicly available (HCP Young Adult—Connectome (humanconnectome.org)).
