# Supplementary material for: Involvement of the ipsilateral-to-the-pain anterior–superior hypothalamic subunit in chronic cluster headache
Source: J Headache Pain. 2024 Jan 11;25(1):7. doi: 10.1186/s10194-023-01711-0 (PMC10782620; doi:10.1186/s10194-023-01711-0)
Supplement: Supplementary file 1 — Additional file 1: Table 1SM. ROI-to-ROI functional connectivity from 166 healthy individuals of the 7T Human Connectome Project (HCP) rs-fMRI dataset for the anterior-superior hypothalamic sub-unit within the areas/structures of the mesocorticolimbic system. Results are significant for parametric multivariate statistics (cluster threshold: p<0.05 cluster-level, p-FDR corrected - MVPA omnibus test; connection threshold: p < 0.05 uncorrected). Abbreviations: hyp ANT-SUP = anterior-superior hypothalamic sub-unit, Medial PFC= medial prefrontal cortex, VTA = ventral tegmental area, R = right, L = left. [file 10194_2023_1711_MOESM1_ESM.docx]

| **ROI to ROI** | | **Statistic** | **p-unc** | **p-FDR** |
| --- | --- | --- | --- | --- |
| Cluster 1/10 |  | F(3,163) = 501.77 | 0.0000 | 0.0000 |
| Amygdala L | Amygdala R | T(165) = 26.28 | 0.0000 | 0.0000 |
| Hippocampus L | Hippocampus R | T(165) = 23.70 | 0.0000 | 0.0000 |
| Amygdala L | Hippocampus L | T(165) = 22.63 | 0.0000 | 0.0000 |
| Amygdala R | Hippocampus L | T(165) = 22.53 | 0.0000 | 0.0000 |
| Amygdala R | Hippocampus R | T(165) = 22.43 | 0.0000 | 0.0000 |
| Amygdala L | Hippocampus R | T(165) = 18.10 | 0.0000 | 0.0000 |
| Cluster 2/10 |  | F(4,162) = 295.30 | 0.0000 | 0.0000 |
| Frontal Pole L | Frontal Orbital L | T(165) = 23.05 | 0.0000 | 0.0000 |
| Frontal Orbital R | Frontal Orbital L | T(165) = 21.86 | 0.0000 | 0.0000 |
| Frontal Orbital R | Frontal Pole R | T(165) = 20.33 | 0.0000 | 0.0000 |
| Frontal Pole L | Frontal Pole R | T(165) = 7.26 | 0.0000 | 0.0000 |
| Cluster 3/10 |  | F(2,164) = 272.31 | 0.0000 | 0.0000 |
| Accumbens R | Accumbens L | T(165) = 19.83 | 0.0000 | 0.0000 |
| VTA R | VTA L | T(165) = 18.99 | 0.0000 | 0.0000 |
| Accumbens R | VTA R | T(165) = 9.68 | 0.0000 | 0.0000 |
| Accumbens L | VTA R | T(165) = 9.11 | 0.0000 | 0.0000 |
| Accumbens R | Hyp. Ant-Sup L | T(165) = 8.74 | 0.0000 | 0.0000 |
| Accumbens R | VTA L | T(165) = 8.64 | 0.0000 | 0.0000 |
| Accumbens L | Hyp. Ant-Sup R | T(165) = 8.05 | 0.0000 | 0.0000 |
| Accumbens R | Hyp. Ant-Sup R | T(165) = 7.58 | 0.0000 | 0.0000 |
| Accumbens L | Hyp. Ant-Sup L | T(165) = 7.54 | 0.0000 | 0.0000 |
| VTA R | Hyp. Ant-Sup L | T(165) = 7.56 | 0.0000 | 0.0000 |
| Accumbens L | VTA L | T(165) = 6.95 | 0.0000 | 0.0000 |
| VTA L | Hyp. Ant-Sup L | T(165) = 6.76 | 0.0000 | 0.0000 |
| Hyp. Ant-Sup R | Medial PFC | T(165) = 6.45 | 0.0000 | 0.0000 |
| Hyp. Ant-Sup L | Medial PFC | T(165) = 5.90 | 0.0000 | 0.0000 |
| VTA L | Hyp. Ant-Sup R | T(165) = 4.17 | 0.0001 | 0.0001 |
| VTA R | Hyp. Ant-Sup R | T(165) = 4.07 | 0.0001 | 0.0001 |
| Cluster 4/10 |  | F(4,162) = 96.89 | 0.0000 | 0.0000 |
| Hyp. Ant-Sup L | Hippocampus R | T(165) = 11.85 | 0.0000 | 0.0000 |
| Hyp. Ant-Sup L | Hippocampus L | T(165) = 11.57 | 0.0000 | 0.0000 |
| Hyp. Ant-Sup R | Hippocampus R | T(165) = 10.86 | 0.0000 | 0.0000 |
| Medial PFC | Amygdala R | T(165) = 10.00 | 0.0000 | 0.0000 |
| Hyp. Ant-Sup R | Hippocampus L | T(165) = 9.82 | 0.0000 | 0.0000 |
| Accumbens L | Hippocampus L | T(165) = 9.50 | 0.0000 | 0.0000 |
| Medial PFC | Amygdala L | T(165) = 9.46 | 0.0000 | 0.0000 |
| Accumbens R | Hippocampus R | T(165) = 9.07 | 0.0000 | 0.0000 |
| Accumbens R | Hippocampus L | T(165) = 8.81 | 0.0000 | 0.0000 |
| Medial PFC | Hippocampus L | T(165) = 8.66 | 0.0000 | 0.0000 |
| Accumbens L | Hippocampus R | T(165) = 8.64 | 0.0000 | 0.0000 |
| Hyp. Ant-Sup L | Amygdala R | T(165) = 8.63 | 0.0000 | 0.0000 |
| Hyp. Ant-Sup L | Amygdala L | T(165) = 7.82 | 0.0000 | 0.0000 |
| VTA R | Hippocampus L | T(165) = 7.52 | 0.0000 | 0.0000 |
| Hyp. Ant-Sup R | Amygdala R | T(165) = 7.52 | 0.0000 | 0.0000 |
| Medial PFC | Hippocampus R | T(165) = 6.97 | 0.0000 | 0.0000 |
| VTA L | Hippocampus L | T(165) = 7.00 | 0.0000 | 0.0000 |
| VTA L | Hippocampus R | T(165) = 6.33 | 0.0000 | 0.0000 |
| Accumbens R | Amygdala L | T(165) = 6.24 | 0.0000 | 0.0000 |
| Hyp. Ant-Sup R | Amygdala L | T(165) = 6.05 | 0.0000 | 0.0000 |
| Accumbens L | Amygdala L | T(165) = 5.43 | 0.0000 | 0.0000 |
| VTA R | Hippocampus R | T(165) = 5.26 | 0.0000 | 0.0000 |
| Accumbens L | Amygdala R | T(165) = 5.12 | 0.0000 | 0.0000 |
| Accumbens R | Amygdala R | T(165) = 4.30 | 0.0000 | 0.0000 |
| VTA R | Amygdala L | T(165) = - 3.01 | 0.0030 | 0.0042 |
| VTA R | Amygdala R | T(165) = - 2.47 | 0.0144 | 0.0168 |
| VTA L | Amygdala R | T(165) = - 2.34 | 0.0207 | 0.0290 |
| Cluster 5/10 |  | F(4,162) = 52.34 | 0.0000 | 0.0000 |
| Frontal Pole R | Amygdala L | T(165) = - 10.93 | 0.0000 | 0.0000 |
| Frontal Pole R | Amygdala R | T(165) = - 7.63 | 0.0000 | 0.0000 |
| Frontal Orbital L | Amygdala L | T(165) = 6.43 | 0.0000 | 0.0000 |
| Frontal Pole R | Hippocampus L | T(165) = - 6.20 | 0.0000 | 0.0000 |
| Frontal Orbital L | Hippocampus L | T(165) = 6.14 | 0.0000 | 0.0000 |
| Frontal Pole R | Hippocampus R | T(165) = - 2.29 | 0.0233 | 0.0297 |
| Frontal Orbital L | Hippocampus R | T(165) = 2.32 | 0.0218 | 0.0436 |
| Cluster 6/10 |  | F(4,162) = 38.56 | 0.0000 | 0.0000 |
| Frontal Pole L | Amygdala R | T(165) = - 10.48 | 0.0000 | 0.0000 |
| Frontal Pole L | Amygdala L | T(165) = - 8.25 | 0.0000 | 0.0000 |
| Frontal Pole L | Hippocampus R | T(165) = - 8.19 | 0.0000 | 0.0000 |
| Frontal Orbital R | Amygdala L | T(165) = - 2.24 | 0.0266 | 0.0532 |
| Cluster 7/10 |  | F(4,162) = 22.62 | 0.0000 | 0.0000 |
| Frontal Orbital R | VTA R | T(165) = 6.77 | 0.0000 | 0.0000 |
| Frontal Orbital R | VTA L | T(165) = 6.36 | 0.0000 | 0.0000 |
| Frontal Pole L | Hyp. Ant-Sup L | T(165) = - 4.90 | 0.0000 | 0.0000 |
| Frontal Pole L | Accumbens R | T(165) = - 4.71 | 0.0000 | 0.0000 |
| Frontal Orbital R | Accumbens L | T(165) = 3.12 | 0.0021 | 0.0050 |
| Frontal Orbital R | Accumbens R | T(165) = 3.12 | 0.0021 | 0.0050 |
| Frontal Pole L | VTA R | T(165) = 2.97 | 0.0034 | 0.0059 |
| Frontal Pole L | Accumbens L | T(165) = - 2.83 | 0.0052 | 0.0081 |
| Frontal Pole L | Medial PFC | T(165) = 2.66 | 0.0085 | 0.0119 |
| Cluster 8/10 |  | F(4,162) = 22.52 | 0.0000 | 0.0000 |
| Frontal Orbital L | Medial PFC | T(165) = 7.59 | 0.0000 | 0.0000 |
| Frontal Pole R | Medial PFC | T(165) = - 4.80 | 0.0000 | 0.0000 |
| Frontal Orbital L | VTA R | T(165) = 4.26 | 0.0000 | 0.0001 |
| Frontal Pole R | Accumbens L | T(165) = - 3.64 | 0.0004 | 0.0007 |
| Frontal Pole R | VTA L | T(165) = 2.92 | 0.0040 | 0.0071 |
| Frontal Pole R | Hyp. Ant-Sup L | T(165) = -2.42 | 0.0165 | 0.0256 |
| Frontal Pole R | Accumbens R | T(165) = - 2.30 | 0.0225 | 0.0297 |
| Cluster 9/10 |  | F(1,165) = 4.09 | 0.0448 | 0.0498 |
| Frontal Orbital R | Frontal Pole L | T(165) = 2.02 | 0.0448 | 0.0785 |

**Table 1SM.** ROI-to-ROI functional connectivity from 166 healthy individuals of the 7T Human Connectome Project (HCP) rs-fMRI dataset for the anterior-superior hypothalamic sub-unit within the areas/structures of the mesocorticolimbic system. Results are significant for parametric multivariate statistics (cluster threshold: p<0.05 cluster-level, p-FDR corrected - MVPA omnibus test; connection threshold: p < 0.05 uncorrected). Abbreviations: hyp ANT-SUP = anterior-superior hypothalamic sub-unit, Medial PFC= medial prefrontal cortex, VTA = ventral tegmental area, R = right, L = left.
